# Identifying the perceptive users for online social systems

**DOI:** 10.1371/journal.pone.0178118

**Published:** 2017-07-13

**Authors:** Jian-Guo Liu, Xiao-Lu Liu, Qiang Guo, Jing-Ti Han

**Affiliations:** 1 Data Science and Cloud Service Research Centre, Shanghai University of Finance and Economics, Shanghai 200433, PR China; 2 Research Center of Complex Systems Science, University of Shanghai for Science and Technology, Shanghai 200093, PR China; 3 Department of Physics, Fribourg University, CH-1700 Fribourg, Switzerland; Beihang University, CHINA

## Abstract

In this paper, the perceptive user, who could identify the high-quality objects in their initial lifespan, is presented. By tracking the ratings given to the rewarded objects, we present a method to identify the user perceptibility, which is defined as the capability that a user can identify these objects at their early lifespan. Moreover, we investigate the behavior patterns of the perceptive users from three dimensions: User activity, correlation characteristics of user rating series and user reputation. The experimental results for the empirical networks indicate that high perceptibility users show significantly different behavior patterns with the others: Having larger degree, stronger correlation of rating series and higher reputation. Furthermore, in view of the hysteresis in finding the rewarded objects, we present a general framework for identifying the high perceptibility users based on user behavior patterns. The experimental results show that this work is helpful for deeply understanding the collective behavior patterns for online users.

## Introduction

Collective behaviors have been extensively investigated to quantitatively explore the behavior patterns of online social systems [1–3], such as the bursty nature, heavy-tailed distribution of inter-event time [4, 5], and so on. Many remarkable patterns and mechanisms of collective behaviors have been proposed, such as the task-based queuing model [6, 7], interest-driven model [8, 9] and radiation model [10], which are gradually applied in the rumor spreading [11, 12], disease spreading [13–15] and recommendation systems [16–18], etc.

Recently, the online user behavior patterns have attracted more and more attention [19–21]. The abundance of available information increases the difficulty in making choices for users: Buy objects, borrow DVDs, or watch movies. Nowadays, online rating systems provide channels for users to show their preferences in the form of ratings [22–24], which can be represented as growing weighted bipartite networks where users are linked with the rated objects over time and the weights are the ratings. Preferential attachment [25, 26], the users connect objects in terms of the object degree preferentially, has been widely used to interpret user rating or selecting behaviors, presenting a homogeneous population composed of users driven by object popularity. Meanwhile, Liu *et al* [17] found that users are heterogeneous in selecting the rated objects: Some objects are collected by almost all users, while some small-degree objects are only collected by large-degree users, indicating that the users’ tastes could be expressed by two categories: Popular one and special one. The work of Ni *et al* [27] also described this idea. Inspired by these work, we carry on investigating the heterogeneity [28–31] of users in their rating patterns. An interesting phenomenon is found: While the majority of users usually collect the popular objects, some users frequently attach to the high-quality objects (which is rewarded finally) when they are rarely concerned, in which the latter group of users are our focus in this paper.

We divide objects into two sets: High-quality objects and the others, in which the high-quality objects are defined as rewarded objects here, e.g. Oscars Award for film, Grammy Award for music, Emmy Award for television, Tony Award for theater, etc. There will be many users focusing on the rewarded objects when they become widely accepted, while an interesting phenomenon is found: There exist some users paying attention to the rewarded objects long before they actually be widely approved (finally rewarded), i.e. at their early lifespan. Here we present two definitions: Perceptive user and user perceptibility. Perceptive user is defined as the user who can make high appraisals of the rewarded objects long before they actually be rewarded. Meanwhile, the degree to which the user can identify the rewarded objects in their initial lifespan is defined as the user perceptibility.

Meanwhile, we present a method to identify the user perceptibility based on online user rating behaviors. Then we investigate the behavior patterns of the perceptive users from three aspects: User activity, correlation characteristics of user rating series and user reputation. Experimental results indicate that high perceptibility users show different behavior patterns than others. Finally, considering the hysteresis in finding the rewarded objects, we present a framework for identifying high perceptibility users based on users’ behavior patterns.

## Material and methods

### Data sets

In this paper, two empirical data sets containing timestamps and ratings for movies: MovieLens and Netflix are investigated. The MovieLens data set is downloaded from the GroupLens (http://www.grouplens.org), consists 943,355 ratings given by 4,295 users to 3,706 movies during 1,039 days. The Netflix data set is provided by the Netflix Prize (http://www.netflixprize.com), consists 37,755,925 ratings delivered by 218,319 users on 7,803 movies during 2,241 days. The MovieLens and Netflix ratings are both given by the integer ratings scaling from 1 to 5. Meanwhile, each user has at least 50 ratings for two data sets. Here, two object sets mentioned above, high-quality objects and the others, are divided based on the Oscars awards. We select movies nominated for the best picture category at the Annual Academy Awards, popularly known as Oscars (http://www.filmsite.org), as the high-quality objects. There are 162 and 150 rewarded movies in the MovieLens and Netflix data sets, respectively.

### Method description

The rating system can be modeled by a weighted bipartite network, where the users and objects are denoted by *U* = {*u*_1_, *u*_2_, …, *u*_|*U*|_}, *O* = {*o*_1_, *o*_2_, …, *o*_|*O*|_}. We use the Latin and Greek letters to represent the users and objects, respectively. The rating *r*_*iα*_ given by user *u*_*i*_ to object *o*_*α*_ is the weight of the link connecting nodes *u*_*i*_ and *o*_*α*_ in the bipartite network. The timestamp of rating *r*_*iα*_ is denoted by *t*_*iα*_ and the highest rating is recorded as *r*_*h*_. The user set *U*_*α*_ is defined as the users who rate to object *o*_*α*_, and the object set *O*_*i*_ is recorded as the objects rated by user *u*_*i*_. In addition, the degrees of user *u*_*i*_ and object *o*_*α*_ are denoted as *k*_*i*_ and *ρ*_*α*_, respectively. Two object sets, rewarded and non-rewarded ones, are denoted by object set *O*_1_ and *O*_2_, respectively, satisfying *O*_1_ ⋃ *O*_2_ = *O* and *O*_1_ ⋂ *O*_2_ = {⌀}. What’s more, the numbers of rewarded and non-rewarded objects are denoted by *n*_1_ and *n*_2_, respectively, *n*_1_ + *n*_2_ = |*O*|.

For each rewarded object, we track the ratings given by users who give the highest rating *r*_*h*_ at the early lifespan of the object. The number of these links *D*_*i*_ created by user *u*_*i*_ can be expressed as,

$$D_{i}=\Sigma_{o_{\alpha }\in O_{1}}D_{i\alpha },$$ (1)

$$D_{i\alpha }=\{\begin{array}{cc} 1 & \text{if}\;r_{i\alpha }=r_{h},t_{i\alpha }\leq t_{\alpha 1}+\left(t_{\alpha \rho_{\alpha }}-t_{\alpha 1}\right)*\theta \\ 0 & \text{else} \end{array},$$ (2)

where *D*_*iα*_ is a binary event to measure whether the user *u*_*i*_ can make a high evaluation of object *o*_*α*_(*o*_*α*_ ∈ *O*_1_) during the initial *θ*(0 < *θ* < 1) of its lifespan, *t*_*α*1_ and *t*_*αρ*_*α*__ are the timestamps of the first and last ratings the object *o*_*α*_ received, respectively. The quantity *D*_*i*_ is the number of identifying rewarded objects at their early lifespan for user *u*_*i*_ and 0 ≤ *D*_*i*_ ≤ *n*_1_. Meanwhile, *θ* is a tunable parameter and the value of *D*_*i*_ increases with the parameter *θ*. It should be noted that there is no rating to be considered (*D*_*i*_ = 0, *i* = 1, 2, …, |*U*|) when *θ* = 0 and the whole lifespan is viewed as the initial lifespan when *θ* = 1.

Finally, we define the perceptibility *p*_*i*_ as the proportion of *D*_*i*_ in the number of rewarded objects *n*_1_ for user *u*_*i*_,

$$p_{i}=D_{i}/n_{1}.$$ (3)

## Results

The identification of the user perceptibility could quantitatively measure the degree to which the user can identify the rewarded objets in their lifepan. To qualitatively measure whether a user is a perceptive user, we a introduce a free-parameter bootstrap analysis [32–34]. The bootstrap sampling results show that, for the MovieLens and Netflix data sets, there are 5 and 27 identified perceptive users, respectively (accounting for 0.12% and 0.012% of all users, respectively). Here the parameter *θ* is set to 0.3 and 0.6 for the MovieLens and Netflix data sets, respectively. It should be noted that a larger parameter *θ* for the Netflix data set is selected due to the few rewarded objects with regard to the size of the whole objects and ratings (150 rewarded objects, 7803 objects and 37755925 ratings).

**Fig 1:**
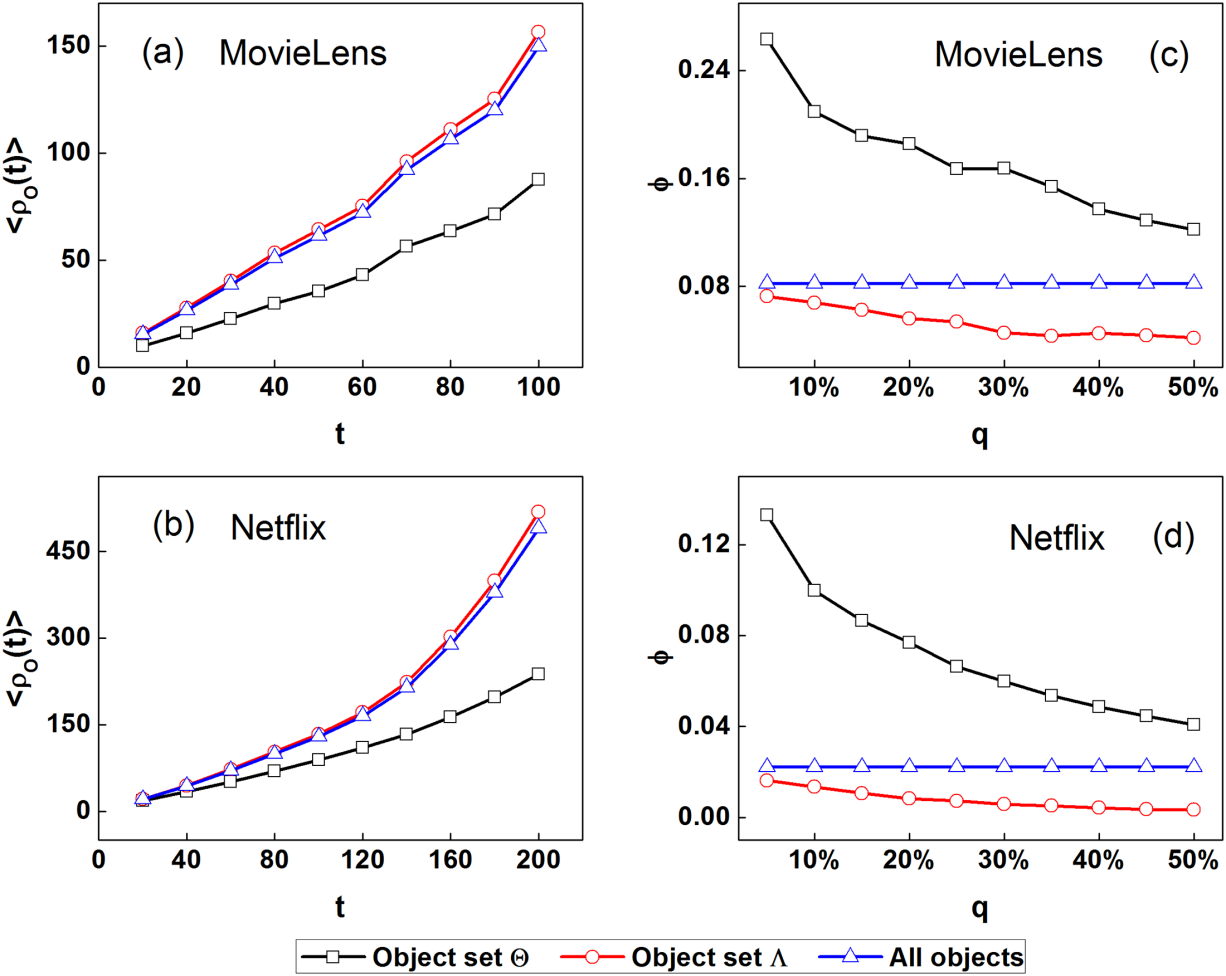
(Color online) Degree evolution 〈*ρ*_*O*_(*t*)〉 of two divided object groups (a,b) and the ratio *ϕ* of rewarded objects in two divided object groups with different parameter *q* (c,d) for two empirical data sets, in which the time *t* is measured in days, and the parameter *θ* is set to 0.3 and 0.6 for the MovieLens and Netflix data sets, respectively. From the subplots (a,b) one can find that the average degrees of objects rated by high perceptibility users in the future time window are larger than those of the other objects. From the subplots (c,d) one can find that the ratio *ϕ* of rewarded objects in objects rated by high perceptibility users is higher than that in the other objects with different parameter *q*. The results indicate that the user perceptibility is helpful to find the potential rewarded objects.

Moreover, we investigate whether the identification of user perceptibility is of significance. To this end, we calculate the average perceptibility of the first *L* users who give the rating 5 (the highest rating) in order of time for each object in two empirical data sets, denoted by 〈*p*^*L*^〉_*α*_ for objects *o*_*α*_. The parameter *L* is set to 10 in the following analysis. All objects are divided into two groups based on their corresponding average perceptibility 〈*p*^*L*^〉: Objects rated by high perceptibility users (recorded as object set Θ) and the others (recorded as object set Λ), in which the objects in set Θ are selected as top *q*(0 < *q* < 1) high 〈*p*^*L*^〉 objects. Firstly, we track the links attached to all objects in the future time window and calculate the average degree 〈*ρ*_*O*_(*t*)〉 of two divided object groups as a function of time *t*, in which the length of the future time window are 100 and 200 days for the MovieLens and Netflix data sets, respectively. Fig 1(a) and 1(b) shows the degree evolution of two divided object groups with the parameter *q* = 10% for the MovieLens and Netflix data sets, respectively. One can find that the average degrees of objects in set Λ in the future time window are larger than those of objects in set Θ, showing that the objects rated by high perceptibility users become less popular than the others, indicating that user perceptibility has little impact on finding the popular objects. Subsequently, we investigate the ratio *ϕ* of rewarded objects in two divided object groups with different parameter *q* (Fig 1(c) and 1(d)). One can find that the ratio *ϕ* of rewarded objects in object set Θ is larger than that in object set Λ with different parameter *q* for two empirical data sets. For instance, the ratio *ϕ* of rewarded objects in object set Θ is larger than that in object set Λ by 263.0% and 722.0% with the parameter *q* = 5% for the MovieLens and Netflix data sets, respectively. Meanwhile, the ratios *ϕ* of rewarded objects in two divided object groups with *θ* = 0.2, 0.4 for MovieLens and *θ* = 0.5, 0.7 for Netflix show the similar results. Therefore, the results indicate that the user perceptibility is of significance in finding the rewarded objects rather than popular objects.

Furthermore, we investigate the relations between user perceptibility and user collective behavior patterns. All users are divided into two groups: High perceptibility users (recorded as user set Φ) and the others (recorded as user set Δ), in which the high perceptibility users are denoted as top *q*(0 < *q* < 1) high perceptibility users. We investigate the collective behavior patterns of two divided user groups from three aspects: User activity, correlation characteristics of user rating series and user reputation. User activity (denoted by *k*_*U*_), namely user degree, is one of the most important user characteristics in social systems [27, 35]. The larger user degree, the more active the user would be. In our analysis, correlation characteristics of user rating series is described by detrended fluctuation analysis (short for DFA), which is widely used for analyzing the statistical self-affinity of a time series [36–39], calculated by the scaling exponent *η*. The quantity *η* > 0: *η* < 0.5 corresponds to anti-correlated series; *η* = 0.5 corresponds to uncorrelated white noise; *η* > 0.5 corresponds to correlated series. User reputation is proposed to measure the user ability of rating accurate assessments of various objects [40, 41]. So far, many reputation ranking methods have been widely investigated [42–44]. In this paper, we use the correlation based ranking algorithm [41] to calculate the user reputation denoted by *μ*. The quantity *μ* lies in [0, 1] and larger *μ* means higher user reputation.

**Fig 2:**
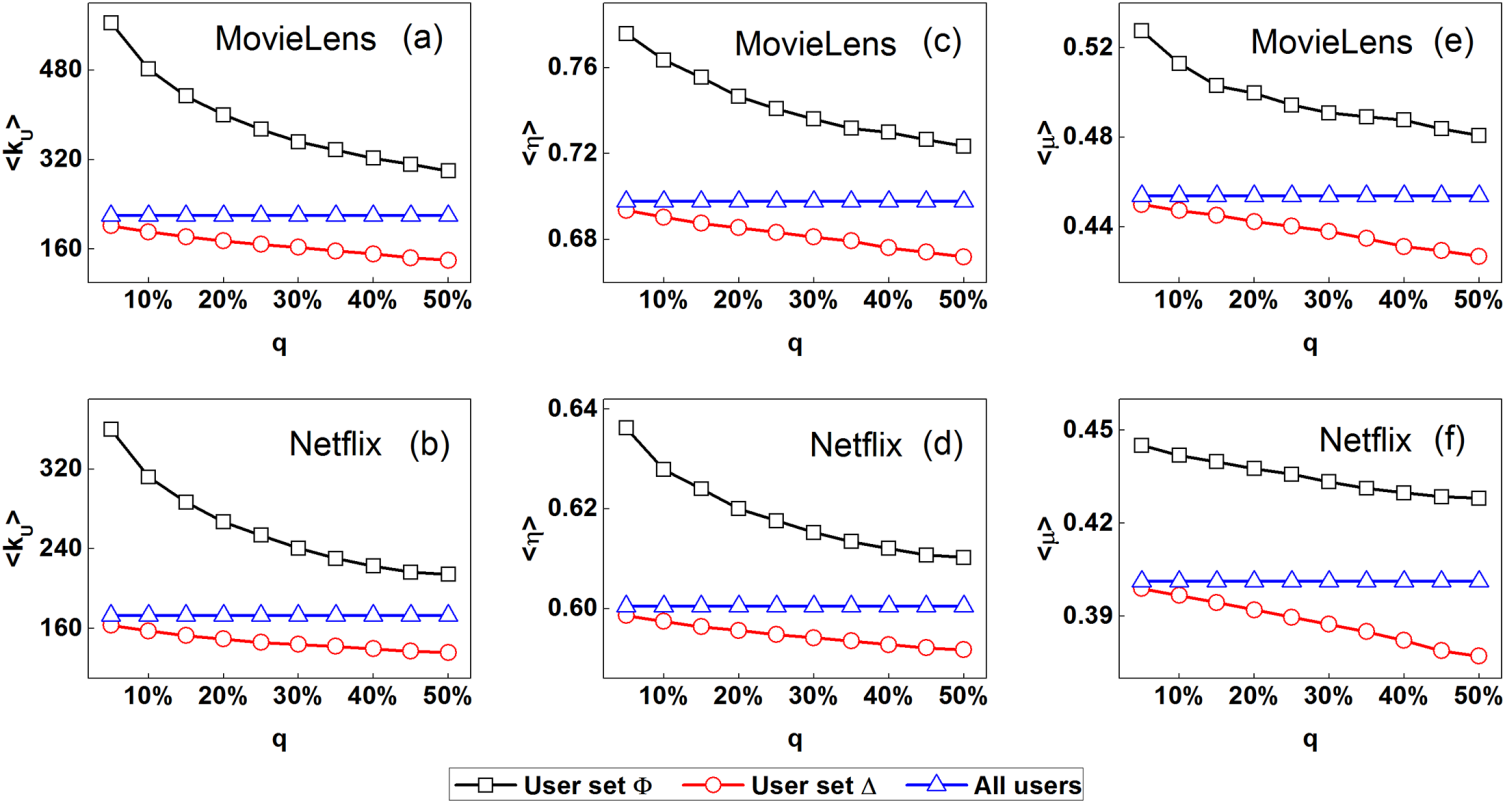
(Color online) The average degree 〈*k*_*U*_〉, scaling exponent 〈*η*〉, reputation 〈*μ*〉 of two divided user groups with different parameter *q* for (a,c,e) MovieLens and (b,d,f) Netflix data sets, in which the parameter *θ* is set to 0.3 and 0.6, respectively. One can find that the average 〈*k*_*u*_〉, 〈*η*〉 and 〈*μ*〉 of high perceptibility users are larger than those of the other users with different parameter *q* for two empirical data sets, which indicates that high perceptibility users show different collective behavior patterns than the other users: Larger activity, stronger correlation of rating series and higher reputation.

Fig 2 shows the average degree 〈*k*_*U*_〉, scaling exponent 〈*η*〉, reputation 〈*μ*〉 of two divided user groups with different parameter *q* for the MovieLens and Netflix data sets, respectively. One can find that the average 〈*k*_*U*_〉, 〈*η*〉 and 〈*μ*〉 of user set Φ (high perceptibility users) are larger than those of user set Δ (the other users) with different parameter *q* for two empirical data sets. For instance, the average 〈*k*_*U*_〉, 〈*η*〉 and 〈*μ*〉 of user set Φ are larger than the ones of user set Δ by 180.1%, 11.8% and 17.3%, respectively with the parameter *q* = 5% for the MovieLens data set. For the Netflix data set, the increases are 120.5%, 6.3% and 11.6%, respectively with the parameter *q* = 5%. The collective behavior patterns of two divided user groups with *θ* = 0.2, 0.4 for MovieLens and *θ* = 0.5, 0.7 for Netflix show the similar results. The results indicate that high perceptibility users show larger activity, stronger correlation of rating series and higher reputation than other users.

## A framework for high perceptibility user identification

High perceptibility users are denoted as top *q*(0 < *q* < 1) high perceptibility users, the identification of high perceptibility users is closely linked with the identification of user perceptibility. User perceptibility is calculated by tracking the ratings to the rewarded objects, while the discovery of the rewarded objects has hysteresis. With the growing amount of new users, objects and the corresponding ratings, the rewarded objects of the current rating systems are uncertain. Thus, the user perceptibility and high perceptibility users cannot be identified in real time. In terms of the fact that high perceptibility users have specific collective behavior patterns, we develop a general framework for identifying high perceptibility users based on users’ behavior patterns.

**Fig 3:**
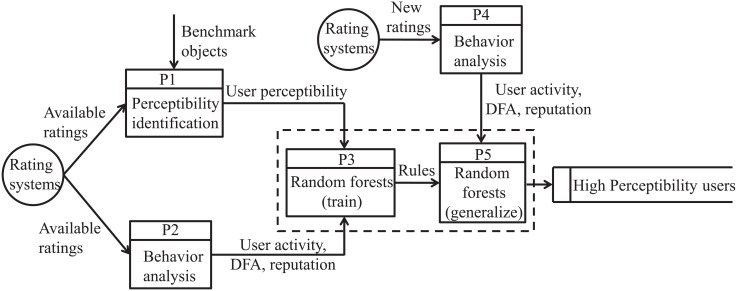
The Data Flow Diagram (DFD) of the framework for high perceptibility user identification. The available ratings in the rating systems, on the one hand, are applied with the rewarded objects to identify the user perceptibility by the presented method (Process P1). On the other hand, they are used to analyze the user collective behavior patterns described by three aspects: Activity, DFA of rating series and reputation (Process P2). Then, we use the random forests to train the obtained results containing the user perceptibility and behavior patterns (Activity, DFA of rating series and reputation) (Process P3). Afterwards, the high perceptibility users will be identified based on the user collective behavior patterns analysed from the new ratings in the rating systems (Process P4) by the generalization of random forests (Process P5).

All users are divided into two groups: High perceptibility users and the others. Given that identifying high perceptibility users belongs to a classification problem, random forests [45], one of the most widely used machine learning [46, 47] methods, is introduced in our framework. The Data Flow Diagram (short for DFD) of the framework is shown in Fig 3. Firstly, the available ratings and the rewarded objects are calculated to identify the user perceptibility using the presented method (Process P1). Meanwhile, the available ratings are used to analyze the user collective behavior patterns from three aspects: Degree, DFA of rating series and reputation (Process P2). The process P1 and P2 could be performed simultaneously. Then, we use the random forests to train the obtained results, which contain the user perceptibility and behavior patterns (Process P3). When the rating systems generate new ratings, the user collective behavior patterns analysed based on the new ratings (Process P4) are used to identify high perceptibility users in the current rating systems by the generalization of random forests (Process P5).

**Fig 4:**
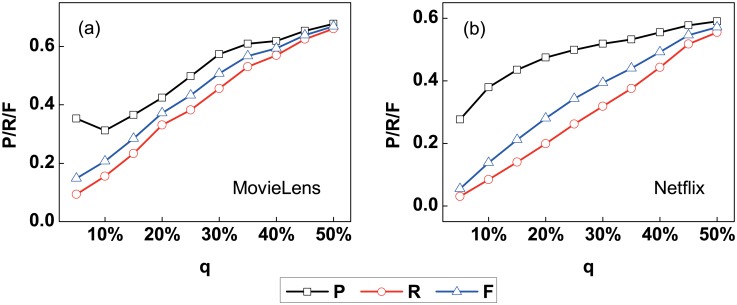
The precision *P*, recall *R* and F-measure *F* of identifying the high perceptibility users in the framework with different parameter *q* for (a) MovieLens and (b) Netflix data sets, respectively. One can find that the precision *P*, recall *R* and F-measure *F* all increase with the parameter *q* in general, and the presented framework can perform well in identifying the high perceptibility users.

Moreover, we investigate the performance of high perceptibility user identification using the presented framework. After identifying the user perceptibility based on the rewarded objects and ratings, high perceptibility users are classified as top *q*(0 < *q* < 1) high perceptibility users. We select 70% of user data (user perceptibility and behavior patterns) as the training set *S*_*tr*_ and the remaining 30% as the test set *S*_*te*_ for the MovieLens and Netflix data sets, respectively. High perceptibility users in the test set *S*_*te*_ are denoted as set *H*_*te*_. Meanwhile, the identified high perceptibility user set $H_{te}^{\prime }$ in the test set *S*_*te*_ will be predicted by the generalization of random forests after training the data of the training set *S*_*tr*_. Then, the performance of high perceptibility user identification is measured by the precision *P*, recall *R* and F-measure *F*,

$$P=\frac{|H_{te}\cap H_{te}^{\prime }|}{|H_{te}^{\prime }|},$$ (4)

$$R=\frac{|H_{te}\cap H_{te}^{\prime }|}{|H_{te}|},$$ (5)

$$F=\frac{2*P*R}{P+R},$$ (6)

where $|H_{te}\cap H_{te}^{\prime }|$ is the number of high perceptibility users in the identified high perceptibility user set $H_{te}^{\prime }$. $|H_{te}^{\prime }|$ is the number of users in the identified high perceptibility user set $H_{te}^{\prime }$. And |*H*_*te*_| is the number of users in the high perceptibility user set *H*_*te*_. Precision *P*, recall *R* and F-measure *F* all lie in [0, 1] and larger *P*, *R* or *F* represents better performance of high perceptibility user identification. The precision *P*, recall *R* and F-measure *F* with different parameter *q* for two empirical data sets are shown in Fig 4, in which the parameter *q*(0 < *q* < 1) represents the ratio of the high perceptibility users in all users. One can find that the framework can perform well in identifying the high perceptibility users. The precision *P*, recall *R* and F-measure *F* could reach *P* = 0.68, *R* = 0.66 and *F* = 0.67 with *q* = 50% for the MovieLens data set, and for the Netflix data set, the performance achieves *P* = 0.59, *R* = 0.55 and *F* = 0.57. Meanwhile, the precision *P*, recall *R* and F-measure *F* all increase with the parameter *q* in general. The performances of high perceptibility user identification with different parameter *θ* indicate that larger precision *P*, recall *R* and F-measure *F* are obtained in the case of larger parameter *θ* with different parameter *q*.

**Fig 5:**
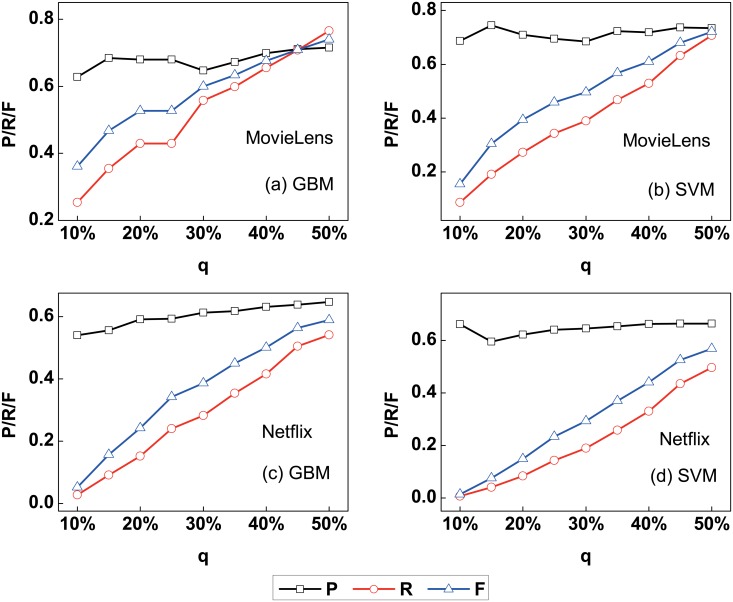
The precision *P*, recall *R* and F-measure *F* of identifying the high perceptibility users in the framework with different machine learning methods (GBM and SVM) and different parameter *q* for (a-b) MovieLens and (c-d) Netflix data sets, respectively. One can find that the recall *R* of high perceptibility user identification using GBM and SVM have little difference with the results using random forests. The precision *P* is better using GBM and SVM than using random forests.

We can obtain both the performance of high perceptibility user identification and importance of behavior patterns using random forests. Besides the random forests, we also use other machine learning methods including gradient boosting machine [48, 49] (short for GBM) and support vector machine [50, 51] (short for SVM) to identify the high perceptibility users. The precision *P*, recall *R* and F-measure *F* of high perceptibility user identification are shown in Fig 5, from which one can find that the performance could reach *P* = 0.72, *R* = 0.77 and *F* = 0.74 using GBM and *P* = 0.74, *R* = 0.71 and *F* = 0.72 using SVM with *q* = 50% for the MovieLens data set. The recall of high perceptibility user identification using GBM and SVM have little difference with the results using random forests. While the precision of high perceptibility user identification using GBM and SVM are different, the precision *P* is large when the parameter *q* is small. The precision is better using GBM and SVM than using random forests.

## Conclusion and discussions

In this paper, taking into account collective behavior patterns and the heterogeneity of online users, we present the definition of perceptive user, which is defined as the user who can make high evaluations of the rewarded objects at their early lifespan. In addition, user perceptibility is defined as the degree to which the user can identify the rewarded objects in their initial lifespan. Then, we present a method for identifying the user perceptibility by tracking the ratings given to rewarded objects and the timestamps. Meanwhile, to track out the relations between user perceptibility and user collective behavior patterns, we investigate the user behavior patterns from three aspects: User activity, correlation characteristics of user rating series and user reputation. The experimental results for the MovieLens and Netflix data sets indicate that high perceptibility users have larger activity, stronger correlation of rating series and higher reputation than the other users. For the MovieLens data set, the average 〈*k*_*U*_〉, 〈*η*〉 and 〈*μ*〉 of user set Φ (high perceptibility users) are larger than those of user set Δ by 180.1%, 11.8% and 17.3%, respectively, with the parameter *q* = 5%, for example. Finally, given that there exists hysteresis in finding the rewarded objects, we present a general framework to identify the high perceptibility users in real time based on users’ behavior patterns. The experimental results show that the framework can perform well in identifying the high perceptibility users. The precision *P*, recall *R* and F-measure *F* could reach *P* = 0.72, *R* = 0.77 and *F* = 0.74 with *q* = 50% for the MovieLens data set, for example.

The computational complexity of the method presented to identify the user perceptibility is *O*(*n*_1_ ⋅ 〈*ρ*_*O*_〉 + *n*_1_ ⋅ |*U*|), where the first term accounts for the calculation of *D*_*iα*_, the results whether the user can make a high evaluation for each rewarded object in its initial lifespan. And the second term accounts for the calculation of *D*_*i*_, the number of identifying the rewarded objects at their early lifespan for each user. Substituting the inequality 〈*ρ*_*O*_〉 ≤ |*U*|, we are left with *O*(*n*_1_ ⋅ |*U*|). Due to *n*_1_ is a constant value in a certain rating system, one has the fact that the computational complexity of the user perceptibility identification is *O*(|*U*|), a linear function of the user size.

For a long time, popular objects are more concerned targets, while few users recognize and appreciate the rewarded objects when they are rarely concerned. The discovery of perceptive users and the identification of user perceptibility provides us a new perspective of understanding these special users. The results that user perceptibility can be helpful to find the potential rewarded objects indicate the identification of user perceptibility is of practical significance in e-commerce and marketing. Meanwhile, the presented framework for high perceptibility user identification, from investigating behavior patterns of two divided user groups to conversely identifying high perceptibility users based on the behavior patterns, gives us a systematic study of the perceptive users and it is also suitable for big data processing. In addition, the following points should be addressed in the future work. Firstly, the high-quality objects here are generated based on the rewarded ones, how to construct the high-quality object set is an open problem. Secondly, the user collective behavior patterns are investigated from three aspects in this paper, which may be found incompletely. As further improvement, we could consider more dimensions to deeper explore the user behavior patterns. Thirdly, random forests is applied in the framework for high perceptibility user identification, in which econometrics and time series analysis could be emphasized as well in our future research.

## Supporting information

S1 Text The bootstrap analysis. (TEX)

S2 Text The ratio *ϕ* of benchmark objects in two divided object groups with different parameter *θ*. (TEX)

S3 Text The calculation process of DFA of user rating series. (TEX)

S4 Text The calculation process of user reputation. (TEX)

S5 Text The collective behavior patterns of two divided user groups with different parameter *θ*. (TEX)

S6 Text The identification performances of high perceptibility users with different parameter *θ*. (TEX)

S7 Text The importance of behavior patterns in random forests. (TEX)

S8 Text The identification performances of high perceptibility users with RBPD reputation ranking method. (TEX)

S1 Fig (Color online) Zipf plots of user perceptibility in real data and bootstrap analysis for (a) MovieLens and (b) Netflix data sets, in which the parameter *θ* is set to 0.3 and 0.6, respectively. (EPS)

S2 Fig The ratio *ϕ* of benchmark objects in two divided object groups with different parameter *q* for (a-b) MovieLens (*θ* = 0.2, *θ* = 0.4) and (c-d) Netflix (*θ* = 0.5, *θ* = 0.7) data sets, respectively. (EPS)

S3 Fig The average degree 〈*k*_*U*_〉, scaling exponent 〈*η*〉, reputation 〈*μ*〉 of two divided user groups with different parameter *q* in the case of *θ* = 0.2, *θ* = 0.4 for the MovieLens data set. (EPS)

S4 Fig The average degree 〈*k*_*U*_〉, scaling exponent 〈*η*〉, reputation 〈*μ*〉 of two divided user groups with different parameter *q* in the case of *θ* = 0.5, *θ* = 0.7 for the Netflix data set. (EPS)

S5 Fig The precision *P*, recall *R* and F-measure *F* of identifying high perceptibility users in the framework with different parameter *q* for (a-b) MovieLens (*θ* = 0.2, *θ* = 0.3, *θ* = 0.4) and (c-d) Netflix (*θ* = 0.5, *θ* = 0.6, *θ* = 0.7) data sets, respectively. (EPS)

S6 Fig The precision *P*, recall *R* and F-measure *F* of identifying the high perceptibility users in the framework with different parameter *q* for (a) MovieLens and (b) Netflix data sets, respectively. (EPS)

S1 Table The importance of user behavior patterns in random forests for the MovieLens data set. (TEX)

S2 Table The importance of user behavior patterns in random forests for the Netflix data set. (TEX)

S3 Table The importance of user behavior patterns in random forests for the MovieLens data set. (TEX)

S4 Table The importance of user behavior patterns in random forests for the Netflix data set. (TEX)

## References

[1] Oliveira J G, Barabási A L. Human dynamics: Darwin and Einstein correspondence patterns. Nature. 2005; 437: 1251–1252. 10.1038/4371251a 16251946

[2] Muchnik L, Aral S, Taylor S J. Social influence bias: a randomized experiment. Science. 2013; 341: 647–651. 10.1126/science.1240466 23929980

[3] Wu Y, Zhou C, Xiao J, Kurths J, Schellnhuber HJ. Evidence for a bimodal distribution in human communication. Proceedings of the National Academy of Sciences. 2010; 107: 18803–18808. 10.1073/pnas.1013140107 PMC2973857 20959414

[4] Barabási A L. The origin of bursts and heavy tails in human dynamics. Nature. 2005; 435: 207–211. 10.1038/nature03459 15889093

[5] Malmgren R D, Stouffer D B, Motter A E, Amaral L A. A Poissonian explanation for heavy tails in e-mail communication. Proceedings of the National Academy of Sciences. 2008; 105: 18153–18158. 10.1073/pnas.0800332105 PMC2587567 19017788

[6] Vázquez A. Exact results for the Barabási model of human dynamics. Physical Review Letters. 2005; 95: 248701 10.1103/PhysRevLett.95.248701 16384430

[7] Vázquez A, Oliveira JG, Dezsö Z, Goh K I, Kondor I, Barabási. Modeling bursts and heavy tails in human dynamics. Physical Review E. 2006; 73: 036127 10.1103/PhysRevE.73.036127 16605618

[8] Han X P, Zhou T, Wang B H. Modeling human dynamics with adaptive interest. New Journal of Physics. 2008; 10: 073010 10.1088/1367-2630/10/7/073010

[9] Zhao Z D, Yang Z, Zhang Z, Zhou T, Huang Z G, Lai Y C. Emergence of scaling in human-interest dynamics. Scientific Reports. 2013; 3: 3472 https://arxiv.org/abs/1307.7796v1 10.1038/srep03472 24326949 PMC3858797

[10] Simini F, González MC, Maritan A, Barabàsi A L. A universal model for mobility and migration patterns. Nature. 2012; 484: 96–100. 10.1038/nature10856 22367540

[11] Kitsak M, Gallos L K, Havlin S, Liljeros F, Muchnik L, Stanley H, et al Identification of influential spreaders in complex networks. Nature Physics. 2010; 6: 888–893. 10.1038/nphys1746

[12] Moreno Y, Nekovee M, Pacheco A F. Dynamics of rumor spreading in complex networks. Physical Review E. 2004; 69: 066130 10.1103/PhysRevE.69.066130 15244690

[13] Pastor-Satorras R, Vespignani A. Epidemic spreading in scale-free networks. Physical Review Letters. 2001; 86: 3200 10.1103/PhysRevLett.86.3200 11290142

[14] Weng L, Menczer F, Ahn Y Y. Virality prediction and community structure in social networks. Scientific Reports. 2013; 3: 2522 10.1038/srep02522 23982106 PMC3755286

[15] Liu J G, Lin J H, Guo Q, Zhou T. Locating influential nodes via dynamics-sensitive centrality. Scientific Reports. 2016; 6: 21380 10.1038/srep21380 26905891 PMC4764903

[16] Zhou T, Kuscsik Z, Liu J G, Medo M, Wakleing J R, Zhang Y C. Solving the apparent diversity-accuracy dilemma of recommender systems. Proceedings of the National Academy of Sciences. 2010; 107: 4511–4515. 10.1073/pnas.1000488107 PMC2842039 20176968

[17] Liu J G, Zhou T, Guo Q. Information filtering via biased heat conduction. Physical Review E. 2011; 84: 037101 10.1103/PhysRevE.84.037101 22060533

[18] Liu J G, Hou L, Pan X, Guo Q, Zhou T. Stability of similarity measurements for bipartite networks. Scientific Reports. 2016; 6: 18653 10.1038/srep18653 26725688 PMC4698667

[19] Hou L, Pan X, Guo Q, Liu J G. Memory effect of the online user preference. Scientific Reports. 2014; 4: 6560 https://arxiv.org/abs/1409.4403 10.1038/srep06560 25308573 PMC4194435

[20] Yang Z, Zhang Z K, Zhou T. Anchoring bias in online voting. Europhysics Letters. 2012; 100: 68002 http://iopscience.iop.org/article/10.1209/0295-5075/100/68002/meta

[21] Ji L, Liu J G, Hou L, Guo Q. Identifying the role of common interests in online user Trust formation. PLoS ONE. 2015; 10: e0121105 10.1371/journal.pone.0121105 26161853 PMC4498922

[22] Zhang Q M, Zeng A, Shang M S. Extracting the information backbone in online system. PLoS ONE. 2013; 8: e62624 10.1371/journal.pone.0062624 23690946 PMC3653959

[23] Zhang Y L, Guo Q, Ni J, Liu J G. Memory effect of the online rating for movies. Physica A. 2015; 417: 261–266. 10.1016/j.physa.2014.09.012

[24] Medo M, Wakeling J R. The effect of discrete vs. continuous-valued ratings on reputation and ranking systems. Europhysics Letters. 2010; 91: 48004 http://iopscience.iop.org/article/10.1209/0295-5075/91/48004/meta

[25] Barabási A L, Albert R. Emergence of scaling in random networks. Science. 1999; 286: 509–512. 10.1126/science.286.5439.509 10521342

[26] Barabási A L. Scale-free networks: a decade and beyond. Science. 2009; 325: 412–413. 10.1126/science.1173299 19628854

[27] Ni J, Zhang Y L, Hu Z L, Song W J, Hou L, Guo Q, et al Ceiling effect of online user interests for the movies. Physica A. 2014; 402: 134–140. 10.1016/j.physa.2014.01.046

[28] Albert R, Jeong H, Barabási A L. Error and attack tolerance of complex networks. Nature. 2000, 406: 378–382. 10.1038/35019019 10935628

[29] Strogatz S H. Exploring complex networks. Nature. 2001, 410: 268–276. 10.1038/35065725 11258382

[30] Kurant M, Thiran P, Hagmann P. Error and attack tolerance of layered complex networks. Physical Review E. 2007, 76: 026103 10.1103/PhysRevE.76.026103 17930100

[31] Motter A E, De Moura A P S, Lai Y C, Dasgupta P. Topology of the conceptual network of language. Physical Review E. 2002, 65: 065102 10.1103/PhysRevE.66.065102 12188771

[32] Efron B. Better bootstrap confidence intervals. Journal of the American statistical Association. 1987; 82: 171–185. 10.2307/2289153

[33] Efron B. Bootstrap methods: another look at the jackknife. Springer New York 1992 http://link.springer.com/chapter/10.1007/978-1-4612-4380-9_41

[34] Athreya K B. Bootstrap of the mean in the infinite variance case. The Annals of Statistics. 1987; 15: 724–731. http://www.jstor.org/stable/2241336 10.1214/aos/1176350371

[35] Liu X L, Guo Q, Hou L, Cheng C, Liu J G. Ranking online quality and reputation via the user activity. Physica A. 2015; 436: 629–636. 10.1016/j.physa.2015.05.043

[36] Peng C K, Buldyrev S V, Havlin S, Simons M, Stanley H E, Goldberger A L. Mosaic organization of DNA nucleotides. Physical Review E, 1994, 49: 1685 10.1103/PhysRevE.49.1685 9961383

[37] Peng C K, Havlin S, Stanley H E, Goldberger A L. Quantification of scaling exponents and crossover phenomena in nonstationary heartbeat time series. Chaos. 1995; 5: 82 10.1063/1.166141 11538314

[38] Rybski D, Buldyrev SV, Havlin S, Liljeros F, Makse H A. Scaling laws of human interaction activity. Proceedings of the National Academy of Sciences. 2009; 106: 12640–12645. 10.1073/pnas.0902667106 PMC2722366 19617555

[39] Rybski D, Buldyrev S V, Havlin S, Liljeros F, Makse H A. Communication activity in a social network relation between long-term correlations and interevent clustering. Scientific Reports. 2012; 2: 560 10.1038/srep00560 22876339 PMC3413962

[40] Laureti P, Moret L, Zhang Y C, Yu Y K. Information filtering via iterative refinement. Europhysics Letters. 2006; 75: 1006 http://iopscience.iop.org/article/10.1209/epl/i2006-10204-8/meta

[41] Zhou Y B, Lei T, Zhou T. A robust ranking algorithm to spamming. Europhysics Letters. 2011; 94: 48002 http://iopscience.iop.org/0295-5075/94/4/48002

[42] Liao H, Zeng A, Xiao R, Ren Z M, Chen D, Zhang Y C. Ranking reputation and quality in online rating systems. PLoS ONE. 2014; 9: e97146 10.1371/journal.pone.0097146 24819119 PMC4018342

[43] Gao J, Dong Y W, Shang M S, Cai S M, Zhou T. Group-based ranking method for online rating systems with spamming attacks. Europhysics Letters. 2015; 110: 28003 http://iopscience.iop.org/0295-5075/110/2/28003

[44] Liu X L, Liu J G, Yang K, Guo Q, Han J T. Identifying online user reputation of user object bipartite networks. Physica A. 2017; 467: 508–516. 10.1016/j.physa.2016.10.031

[45] Breiman L. Random forests. Machine learning. 2001; 45: 5–32.

[46] Goldberg D E, Holland J H. Genetic algorithms and machine learning. Machine learning. 1988; 3: 95–99. 10.1007/BF00113892

[47] Cortes C, Vapnik V. Support-vector networks. Machine learning. 1995; 20: 273–297. 10.1007/BF00994018

[48] Friedman J H. Greedy function approximation: a gradient boosting machine. Annals of Statistics. 2001, 29: 1189–1232. http://www.jstor.org/stable/2699986

[49] Friedman J H. Stochastic gradient boosting. Computational Statistics & Data Analysis. 2002, 38: 367–378. 10.1016/S0167-9473(01)00065-2

[50] Suykens J A K, Vandewalle J. Least squares support vector machine classifiers. Neural Processing Letters, 1999, 9: 293–300. 10.1023/A:1018628609742

[51] Furey T S, Cristianini N, Duffy N, Bednarski D, Schummer M, Haussler D. Support vector machine classification and validation of cancer tissue samples using microarray expression data. Bioinformatics. 2000, 16: 906–914. 10.1093/bioinformatics/16.10.906 11120680

